# In their own words: A qualitative study of patient narratives on daily life after breast cancer radiotherapy

**DOI:** 10.1016/j.pecinn.2026.100468

**Published:** 2026-03-06

**Authors:** Madeline S. Therrien, Simone M.B. Mingels, Rianne R.R. Fijten, Daniela B. Raphael, Marcel Stam, Desiree van den Bongard, Olga C. Damman, Liesbeth J. Boersma

**Affiliations:** a Department of Radiation Oncology (Maastro), GROW Research Institute for Oncology and Reproduction, Maastricht University Medical Centre +, Maastricht, the Netherlands; b Amsterdam UMC, Department of Radiation Oncology, Amsterdam, the Netherlands; c Radiotherapiegroep, Arnhem, the Netherlands; d Cancer Center Amsterdam, Cancer Treatment and Quality of Life, Amsterdam, the Netherlands; e Department of Public and Occupational Health, AmsterdamUMC, Location VUmc, Amsterdam, the Netherlands; f Amsterdam Public Health Research Institute, Quality of Care, Amsterdam, the Netherlands

**Keywords:** Breast neoplasms, Cancer, Decision support techniques, Oncology, Patient-centered care, Personal narratives, Qualitative research

## Abstract

**Objectives:**

To explore how former breast cancer patients (BCPs) experience the daily-life impact of radiotherapy (RT) side effects and to examine how these narratives can inform the selection of patient stories for the BRASA patient decision aid (BRASA-PtDA).

**Methods:**

Semi-structured interviews were conducted with fourteen former BCPs who completed adjuvant RT 6 months–5 years earlier. Thematic analysis identified locoregional, general, and overarching impacts on quality of life (QoL). Narrative excerpts were classified by type, purpose, and evaluative valence.

**Results:**

Participants described locoregional side effects (pain, reduced arm function, changes in breast appearance, skin irritation) and general effects (fatigue, concentration difficulties) affecting daily activities and self-perception. Three overarching themes—comparisons to the pre-treatment self, acceptance, and work—captured broader QoL impacts. All narratives were experiential or outcome-focused, mostly negative in valence. Thirteen balanced narratives were selected for potential inclusion in the BRASA-PtDA.

**Conclusions:**

Narratives from BCPs illustrate how RT side effects shape daily life and overall QoL. Structured narrative classification provided a transparent method for selecting stories for a decision aid. Future research should examine how different narrative types influence patient engagement and decision outcomes.

**Innovation:**

To our knowledge, this is the first qualitative study to examine how BCPs live with RT side effects, highlighting impacts on daily life that are under-communicated in current practice in the Netherlands and internationally. Findings provide rich insights for improving risk communication and shared decision making by moving beyond symptom-focused information to incorporate patients' lived experiences.

## Highlights

• Explored daily-life impacts of breast cancer radiotherapy side effects.
• Identified cross-cutting QoL themes: acceptance, work, and comparisons to pre-treatment self.
• Applied deductive thematic analysis using a predefined codebook.
• Classified patient stories via the Taxonomy of Patient Narratives in Decision Aids.
• Informed narrative selection for the BRASA-PtDA to support shared decisions.

## 1 Introduction

In the Netherlands, approximately 67% of patients with invasive non-metastatic breast cancer receive radiotherapy (RT) [1]. Similar rates are reported elsewhere: roughly 65% in the UK and 70% in Canada and the USA [2], [3], [4], [5]. However, sometimes, RT reduces local recurrence without substantially altering survival [6], making the decision to undergo RT highly preference-sensitive especially considering potential short- and long-term side effects. While RT's clinical effectiveness and side effects are well documented, less is known about how side effects affect daily life and overall quality of life (QoL), which is crucial for enhancing care and supporting shared decision-making (SDM).

SDM is a process in which healthcare providers (HCPs) and patients jointly determine the most appropriate treatment based on evidence and patient preferences [7]. It involves four steps, where the HCP: (1) informs the patient that a decision is needed; (2) explains care options with pros and cons; (3) supports deliberation and discusses preferences; and (4) with the patient makes or defers the decision [8]. After SDM, patients are more likely to feel satisfied with their treatment decision, and experience less decisional conflict [9].

To support SDM for breast cancer RT in the Netherlands, the BRASA Patient Decision Aid (BRASA-PtDA) was developed [10], [11]. This online tool provides evidence-based information about RT, including its impact on tumor recurrence and potential side effects [12]. After launching the BRASA-PtDA, the developers learned that users wanted the tool to illustrate how side effects may impact daily life, rather than focusing only on their statistical likelihood [13], [14]. This feedback aligns with prior research showing that breast cancer patients (BCPs) consistently seek more information about the QoL consequences of treatment side effects and their impact on everyday functioning [11], [14], [15], [16].

The use of patient narratives is one way to help convey potential QoL impacts in PtDAs, but this practice remains controversial. While narratives may enhance engagement with the information presented, they may influence decisions more strongly than statistical data, raising concerns about overvaluing individual stories over broader evidence. Therefore, experts stress the importance of carefully selecting narrative types and tones to minimize these risks [17], [18].

To navigate this process, Shaffer and Zikmund-Fisher proposed the Taxonomy of Patient Narratives in Decision Aids, which emphasizes evaluating the purpose, content, and evaluative valence of patient narratives to predict their impact on decision making. The taxonomy acknowledges that different narrative types may influence patients' perceptions and choices in distinct ways and offers a conceptual framework to guide narrative selection in PtDAs. However, its application to real-world patient narratives has not yet been empirically validated [18].

To address both the communication gap identified by BCPs regarding the daily-life impact of RT and the need to better understand how patient narratives can be meaningfully incorporated into PtDAs, this study explores how former BCPs experience RT side effects. These insights are expected to help identify QoL aspects that warrant greater attention during SDM consultations. In parallel, we apply the Taxonomy of Patient Narratives in Decision Aids in categorizing narratives and to inform narrative selection for the BRASA-PtDA, while also assessing its practical utility.

## 2 Methods

We conducted a qualitative study employing thematic analysis based on semi-structured interviews guided with a pre-defined interview guide (Appendix A) with former BCPs who had undergone RT. In addition to exploring how BCPs experience RT side effects, we classified the narratives using the Taxonomy of Patient Narratives in Decision Aids [17]. The study is reported following the Consolidated Criteria for Reporting Qualitative Research (COREQ) checklist [19] (Appendix B).

### 2.1 Research team

All interviews were conducted by MT, MSc (Master of Science). MT are her initials. She is a PhD student at Maastro (the Netherlands) with training in qualitative methods, including interview techniques gained during her academic studies. She had no prior relationship with any of the participants. Before each interview, MT introduced herself by sharing her background and explained her professional interest in health communication and SDM.

### 2.2 Ethics approval

The protocol of this study was approved by Maastro's Institutional Review Board (IRB) (identifier: W 24 0100029) and the Medical Ethics Review Committee (METC) of Maastricht University Medical Centre (MUMC+). The METC declared the study to be non-WMO (non-medical research involving human subjects under Dutch law), meaning that it did not require formal medical ethics approval as per the Dutch Medical Research Involving Human Subjects Act (identifier: METC 2024–0081).

### 2.3 Study design

#### 2.3.1 Participant selection

We aimed to conduct 10–15 semi-structured qualitative interviews with former BCPs who had undergone RT. Participants were selected using purposive sampling and had to meet the following inclusion criteria: (a) Completion of whole breast RT (with or without a boost), partial breast or chest wall RT within 6 months to 5 years prior to the interview; (b) Willingness to participate in an interview and share personal experiences; (c) Ability to communicate verbally in English or Dutch. There were no explicit exclusion criteria beyond failure to meet the above.

Recruitment took place via two routes: (1) At hospitals in Maastricht (Maastro), Arnhem (Radiotherapiegroep), and Amsterdam (Amsterdam University Medical Centre), clinicians identified and approached eligible patients based on the inclusion criteria. After providing study information, clinicians obtained permission for MT to contact patients directly; (2) An open call posted on Maastro's patient panel, inviting interested individuals to contact MT.

Informed consent was obtained prior to the interviews, with MT available to answer any questions. Basic demographic information was collected at the beginning of each interview.

This study did not aim for data saturation; instead, our objective was to gather narratives that reflected the key themes identified in Roumen et al. (2022) [13], which are already addressed in the BRASA-PtDA. We sought to capture multiple perspectives for each theme to inform the development of patient narratives for the BRASA-PtDA. Additional narratives beyond these themes were welcome but not required.

#### 2.3.2 Thematic analysis

This study followed principles of thematic analysis (Braun & Clarke, 2006) [20], applied in a deductive manner using a predefined code book (See Appendix C). Initial codes were derived from Roumen et al. (2022) [13], ensuring that the analysis was grounded in known RT side effects and related QoL impacts. While the phases of thematic analysis suggested by Braun and Clarke were not followed in a fully inductive way, the process retained their emphasis on systematic coding, theme refinement, and transparency.

We used Braun and Clarke's thematic analysis to identify patterns across participants regarding QoL impacts, directly informing decision aid development. However, to resolve ‘hybrid’ narratives that straddled taxonomy categories, we also drew on Riessman's thematic narrative analysis [21] by considering the broader narrative context to identify the dominant theme of an account. This ensured our content remained representative of the patient experience while meeting the structured requirements of PtDA development.

Interviews were audio-recorded and transcribed verbatim; transcripts were not returned to participants for comment. Coding and analysis were performed on the original Dutch transcripts, with selected quotes later translated into English by MT for reporting. Transcripts were coded in Atlas.ti (v25.0.1).

Initially, MT coded the data. Later, a second researcher, SM, independently coded three interviews to check consistency. Coding differences were discussed by MT and SM and resolved through consensus, enhancing the credibility and rigor of the analysis. During coding, some QoL impacts were found to cut across multiple side effects and were added as separate themes. A subtheme was included only if described by at least two participants. Participants did not provide feedback on the findings.

#### 2.3.3 Analysis of narratives

Narrative excerpts from the thematic analysis were categorized using the Taxonomy of Patient Narratives in Decision Aids [14]. This defines three narrative dimensions that may influence decision making: purpose, content, and evaluative valence. Purpose reflects the narrative's function—e.g., to inform, engage, model behavior, persuade, or comfort. For the BRASA-PtDA, the focus was on narratives that inform and engage.

Content was categorized as outcome (describing health outcomes associated with a treatment decision), experiential (illustrating what it feels like to undergo treatment or live with a side effect), or process (detailing how a treatment decision was made). Narratives with multiple elements were classified by their primary emphasis. For example, a narrative was coded as experiential if the focus remained on daily management or coping, even when clinical outcomes were mentioned. This ensured each excerpt was categorized based on its dominant meaning.

Each narrative was analyzed by MT for content type and evaluative valence, rated on a 5-point Likert scale from very positive to very negative. Independent coding by multiple researchers was not conducted, as these ratings were used to guide narrative selection rather than for hypothesis testing or statistical analysis.

## 3 Results

Fourteen biologically female participants (mean age = 56.9 years; range = 35–72) were included. Most were employed (six part-time, three full-time), and five were not working (retired or unemployed) (Table 1). All had completed RT for breast cancer between six months and five years prior. Demographic information was limited to age, employment status, and sex. Age and sex were included for their relevance to treatment preferences and QoL, and employment status for its relation to work impacts. Other variables were not systematically collected.

**Table 1:** Participant demographics.

Participant	Age	Sex	Working status
1	72	Female	Retired
2	66	Female	Retired
3	35	Female	Unemployed
4	64	Female	Employed (full time)
5	42	Female	Employed (part time)
6	40	Female	Employed (part time)
7	68	Female	Retired
8	55	Female	Employed (part time)
9	59	Female	Unemployed
10	54	Female	Employed (part time)
11	64	Female	Employed (full time)
12	62	Female	Employed (part time)
13	63	Female	Employed (full time)
14	53	Female	Employed (part time)

Interviews took place privately online or at Maastro, depending on participant preference, and were conducted in Dutch. Most often, only MT and the participant were present; two participants were accompanied by a family member. There were no dropouts, refusals, or repeat interviews. Sessions lasted 25–60 min.

### 3.1 Impacts of breast cancer RT on daily life

To structure the analysis, we used three categories: 1) Locoregional side effects; 2) General side effects; and 3) Overarching impacts. Within the first two categories, side effects were coded as subcategories, and the associated themes reflected impacts that were unique to a single side effect. The final category, overarching impacts, captured broader QoL impacts discussed by participants in relation to multiple side effects.

#### 3.1.1 Category 1: Locoregional side effects

Participants reported a range of locoregional side effects following RT, affecting pain, arm functionality, breast appearance, and skin condition. These experiences influenced daily life in multiple ways (See Table 2a).

**Table 2:** a. Overview of results.

Category	Sub-category	Theme	Number of participants
Locoregional side effects	Pain	Pain with touch	7
		Chronic discomfort	5
		Sports	2
	Arm functionality	Limitations in movements	6
		Loss of strength	2
	Changes in breast appearance	Managing a new appearance	4
		Partner support	3
	Skin irritation	Needing aftercare	6
		Unsatisfactory cosmetics	3
		Sun avoidance	3
		Difficulties with intimacy	2
General side effects	Fatigue	Fluctuating energy	5
		Stamina	3
	Concentration	Difficulty reading	2


b. Overview of results			
Category	Theme	Side effect	Number of participants
Overarching impacts	Comparing to pre-treatment self	Changes in breast appearance	3
		Fatigue	4
		Concentration	3
	Acceptance	Pain	2
		Changes in breast appearance	3
	Work	Fatigue	6
		Concentration	4

##### 3.1.1.1 Pain

Many participants described persistent pain in the irradiated area. Some experienced pain triggered by direct physical contact, such as hugging, lying on the treated side, during medical examinations, wearing a bra, or incidental bumps. Others reported chronic discomfort characterized by ongoing irritation or unease rather than sharp pain.

*“It's not that I can't do certain things, but if someone… if someone hugs me and its too tight, then… ouch” (Participant 9).*

For a small subset (*n* = 2), physical activity such as sports exacerbated this discomfort, limiting their ability to exercise or perform specific movements.

##### 3.1.1.2 Arm functionality

Participants noted limitations in arm mobility, including difficulty reaching, lifting, or twisting. These restrictions affected everyday tasks, such as retrieving items from high cupboards and participating in ball sports. In some cases, decreased arm strength also made tasks like cycling or operating doors challenging.

*“… in some areas I am more often – well, ‘limited’ is such a big word – but you do notice it. For example, if I have to take something out of the cupboard, everything feels really sensitive” (Participant 8).*

##### 3.1.1.3 Changes in breast appearance

Several participants discussed impacts related to altered breast appearance post-treatment. This included managing asymmetry, changes in size, or dissatisfaction with the aesthetic outcome of reconstruction or RT. Clothing choices were sometimes adapted to accommodate these changes. Support from partners emerged as a key coping mechanism, helping participants mitigate the impact of appearance-related concerns.

*“… when I was told I had cancer, my husband came home and, very lovingly, said: ‘Even if they have to remove your breast, I still love you. So don't worry about that – we'll get through this together.’ That meant everything. He had no idea how important that was for me” (Participant 4).*

##### 3.1.1.4 Skin irritation

Skin changes were a commonly reported locoregional effect. Participants described the need for aftercare to manage irritation, such as peeling, scarring, or changes in pigmentation. Some also reported dissatisfaction with the appearance of their skin, which was associated with peeling, dark armpits, white nipples, or a leathery texture. Sun avoidance was another important consideration, requiring caution during outdoor activities to prevent further irritation. Finally, in some cases, these skin changes were also said to affect intimacy due to pain or loss of sensitivity.

*“… when my husband touches my breast, [the irradiated] one is less sensitive. Also the.. yeah, I'm a bit embarrassed to say it… the nipple is less sensitive” (Participant 12).*

#### 3.1.2 Category 2: general side effects

Participants described several side effects that, while not necessarily specific to RT, were experienced during the post-treatment period.

##### 3.1.2.1 Fatigue

Post-treatment fatigue was also commonly reported. Some participants described this as fluctuating energy levels throughout the day, requiring periods of rest to manage sudden dips. These fluctuations were perceived as particularly disruptive when balancing daily responsibilities such as work, household tasks, or social activities. Several participants emphasized that this fatigue felt distinct from ordinary tiredness, describing it as unpredictable and overwhelming.

For others, fatigue was more clearly reflected in reduced stamina. Even small amounts of physical activity—such as walking short distances, cycling, or engaging in rehabilitation exercises—became unexpectedly difficult for them.

*“It was a very strange kind of fatigue… it wasn't the kind of tiredness like, “Oh, I've done a lot, and now I'm tired.” No, it was more like, “Oh…”— it would just overcome you. And then, “Wow, I need to lie down now”. (Participant 2).*

##### 3.1.2.2 Concentration difficulties

Some participants noted difficulty maintaining concentration post-treatment. In particular, two individuals reported that reading required more mental effort than before treatment, leading to frustration and a sense of reduced mental sharpness.

*“But when I read, I really do have to… I actually have to sit down for it, and I really have to be able to concentrate. Because otherwise I'll be reading and at some point I'll think… ‘Wait, what was that again?’ And then I have to go back a page” (Participant 13).*

#### 3.1.3 Category 3: overarching impacts

During our analysis, three overarching impacts on QoL emerged: comparing to pre-treatment self, acceptance, and work. These impacts were discussed in relation to multiple side effects, rather than being unique to just one. For that reason, we present them separately here.

##### 3.1.3.1 Comparisons to pre-treatment self

A recurring thread across interviews was the tendency for participants to evaluate their current post-treatment state against their perception of themselves before RT. This reflective process emerged in relation to changes in breast appearance, fatigue, and concentration. In some cases, these comparisons intensified feelings of loss or frustration. For example, when participants compared their current breast appearance with its pre-treatment state, or when they found themselves unable to match their former levels of physical endurance or ability to focus.

*“Well, you know… sometimes its frustrating. You just want to get back to normal again… You think, damn it. I just want to have the ‘normal’ back a bit” (Participant 8).*

##### 3.1.3.2 Acceptance

For some participants, acceptance appeared to be a coping mechanism. This was most often discussed in the context of breast appearance, with several women explaining an ability to accept this change due to older age or as a trade-off for being cancer free. Acceptance also emerged in relation to pain, where participants described adapting to discomfort rather than resisting it. Although not always framed as a deliberate coping strategy, these accounts suggest that acceptance may be a common way of easing the emotional distress associated with lasting treatment effects.

*“You also learn to… you start accepting things. Eventually, you even start to accept pain” (Participant 12).*

##### 3.1.3.3 Impact on work

Work life was another domain affected, particularly by fatigue and concentration difficulties. Fatigue was reported to limit work performance, with some participants feeling pressured to meet pre-treatment standards despite ongoing symptoms, and/or struggling to communicate their needs for adjustments in their work schedule to colleagues or supervisors. Concentration challenges added to work-related difficulties, with participants describing requiring additional mental effort to focus and, in some cases, leading to strategies such as reducing workplace distractions.

*“Working was really hard. In the evenings, I was completely exhausted. I was really dead tired because I had to put so much effort into maintaining my concentration” (Participant 2).*

### 3.2 Narrative classification by taxonomy

All narratives were classified as either *experiential* or *outcome* and most narratives carried a negative evaluative valence. The most commonly observed narrative type varied by theme: experiential narratives were more common in *pain*, *changes in breast appearance*, *fatigue*, *acceptance*, and *work*; whereas outcome narratives were predominant in: *arm functionality*, *skin irritation*, *concentration*, and *comparing to pre-treatment self*.

#### 3.2.1 Narrative selection

Thirteen narratives were selected for inclusion in the BRASA-PtDA. Eleven were drawn from the thematic analysis, with the exception of two short-term pain narratives, which were mentioned by only one participant each and thus not retained in the analysis (Table 3). Of the included narratives, seven were classified as outcome-based and six as experiential. Most carried a neutral evaluative valence, with four negative and one positive. For the side effect *long-term changes in breast appearance*, we included both a negative outcome narrative and a positive experiential narrative to balance potential steering influence on patients. Overall, narratives with neutral valence were prioritized with the aim that they inform users without exerting undue persuasive effects.

**Table 3:** Selected narratives.

Side Effect		Narratives	Content	Evaluative valence
Short term	Skin	“After my last radiation treatment, let's say about three weeks later, we went on vacation. We went to Greece. I had asked beforehand whether that would be a problem. I just had to be careful, wear a shirt over it and stay in the shade. I had to be mindful of that anyway.”	Outcome	Neutral
		“Yes, I did have some skin complaints. The tissue swelled up. I had some skin issues, so some discoloration appeared. The skin started to peel a little, but that was resolved with an ointment. It went away after a few weeks.”	Outcome	Neutral
	Fatigue	“I talked to my manager, and he said, you know what, just come in, and if you can't do much, then don't do much. But that didn't really work out. So what I want to say is, that was probably the hardest time — going to work when I was tired, having stomach pain, headaches, and maybe also emotional stuff because I was so tired.”	Experiential	Negative
	Pain	“I had pain in my ribs, but after a while that pain went away. Still, it feels a bit sensitive there, even though it doesn't hurt anymore”	Outcome	Neutral
		“During the radiotherapy, I needed to lay with my arms above my head. At one point, I felt pain in my arms, so I put them down. Then the assistant said that I had to start all over again.”	Experiential	Negative
Long term	Changes in breast appearance	“Everything on that side just looks different. So yeah, it's different for everyone, but I've simply accepted it — and again, my sweet husband as well. That really makes a big difference overall. So for me, yes, the difference is there, but… well, the cancer is gone.”	Experiential	Positive
		“So you see, for example… I used to have round breasts. Like apples. And this one was corrected and has stayed like an apple. But this one—there's nothing left here. It was also located here, so they had to do all kinds of tricks and techniques to make it look somewhat decent. And with the scar—I still have a hard patch here, a firm edge. And they can't do anything about it, because according to the oncology team and the plastic surgeon, it's a result of the radiation, which caused it to harden.”	Outcome	Negative
	Reduced arm function	“But you know, I've always had an office job. And now I'm retired. So I can completely adjust my movements to suit myself. But if I still had a different kind of job now — one where you really need to reach a lot and use your arms — then I think I would definitely be struggling with it.” (Moderator: And that's different because you're retired now?) “Yes, now I don't have to. It's like taking the elevator instead of the stairs — that's how I see it. I can still take the stairs, sure. But why would I? That's how I look at it.”	Outcome	Neutral
		“Yes, when I'm exercising, I definitely notice it. With certain exercises, that shoulder is limited. They're really strange movements, because if I just calmly raise my arm, that's fine. But if I have to make an unexpected movement, especially a twisting one—like a front crawl in the pool—that just doesn't work. But just swimming is okay.”	Outcome	Neutral
	Pain and sensitivity	“Yes, it basically turns into light scar tissue and becomes hard, and if someone hugs you a bit too firmly, then you feel that — and that doesn't go away, I still have that. But that probably won't ever go away.”	Outcome	Neutral
		“You never normally feel this. Normally, you don't feel your limbs at all. Not even when you move them. But now you do feel them. They're basically present all day—sometimes more, sometimes less, but they're always there. And you feel that.”	Experiential	Neutral
	Fatigue	“No, I don't really avoid anything. I still have young children and, well, just a family. And that's what keeps you going. But if we have, say, a party or something in the evening, then I'll make sure to take a proper nap in the afternoon, and that works out fine. So no, it's not a problem at all.”	Experiential	Neutral
		“That… immense fatigue, it's not regular tiredness. Like after a day of work — no, it's a kind of exhaustion that just knocks you out. And it hits you at moments when you're not really expecting it. One day you're fine, and the next… Even now, I still experience a certain fatigue — like behind my eyes. But then I wonder, is it hay fever? You never really know what exactly causes it. I still sleep almost every day.”	Experiential	Negative

## 4 Discussion and conclusion

### 4.1 Discussion

#### 4.1.1 Main findings

The primary aim of this study was to explore how former breast cancer patients (BCPs) experience radiotherapy (RT) side effects in daily life. Two pre-defined categories were used to guide our analysis—locoregional side effects, general side effects—within which we examined six side effects of interest: pain, arm function, changes in breast appearance, skin irritation, fatigue, and concentration. Our findings illustrate how these physical effects translated into concrete disruptions of experienced daily life and overall QoL.

Both locoregional and general side effects of breast cancer RT had substantial impacts on participants' daily lives and overall QoL. These impacts varied by side effect as well as between individuals, but the main findings were consistent in showing that the consequences extended well beyond the treatment period. In addition to these specific side effects, our analysis identified three overarching impacts on QoL: comparisons to pre-treatment self, acceptance, and work. While other impacts were tied to specific symptoms, these three themes functioned as cross-cutting issues that emerged across multiple different side effects.

#### 4.1.2 Discussing impacts in SDM consultations

Discussing the impact of treatment on QoL is essential for step 2 of the SDM process. In step 2, the HCP explains the care options, and their pros and cons, to the patient [8]. Our interviews revealed a variety of impacts, with recurring themes, providing guidance for clinicians on which QoL topics to discuss. For example, when discussing concentration as a potential side effect, clinicians may ask patients if reading is one of their hobbies; if so, they could explore how post-treatment concentration difficulties might affect this activity, and whether this is important to the patient. These discussions can help tailor care to patients' preferences and build trust [22], [23], [24].

In addition, providing patients with this information during the consultation and through evaluated tools, such as the BRASA-PtDA, ensures that they receive more reliable information than what they might find online. This is important, as research shows that most BCPs seek information about their treatment on the internet to fill gaps in the information they have received [25]. However, studies indicate that the overall quality of available online resources on breast cancer varies widely [25], and finding reliable online resources can be challenging [26].

#### 4.1.3 Selecting narratives for the BRASA-PtDA

The Taxonomy of Patient Narratives offered a structured, evidence-informed approach to guide narrative inclusion in the BRASA-PtDA. All narratives were classified as either experiential or outcome-based. Process narratives did not emerge, likely because the interviews focused on participants' experiences with the side effects of RT rather than on their decision-making processes.

The taxonomy was a useful guide, but the line between ‘experience’ and ‘outcome’ narratives was sometimes blurry. In these cases, we drew on Riessman's thematic narrative approach [21] by looking at the narrative as a whole to identify the primary thematic focus. For example, a patient might mention a physical side effect but frame it primarily through its impact on daily life, making the account more experiential than outcome-based. This suggests that PtDA developers need a degree of narrative awareness to interpret how patients integrate these elements and classify them by their primary focus*.* The taxonomy's developers also noted these overlaps [19] and further research is needed to see how these ‘hybrid’ narratives influence patient decision-making.

#### 4.1.4 Limitations

Several limitations should be considered. All participants were former BCPs from three hospitals in the Netherlands, which may limit generalizability to other populations or healthcare settings. Demographic information was limited to age, sex, and employment status; other potentially relevant factors (e.g., additional treatments, stage of disease, race, education, or comorbidities) were not systematically collected, which may constrain interpretation of the findings. While the interviewer aimed for an open, non-judgmental environment, participants' stories may reflect conscious or unconscious framing, and self-reported narratives are shaped by memory and personal interpretation. Additionally, field notes were not systematically collected during data collection, which may have limited the depth of contextual and reflective insights. Also, while the Taxonomy of Patient Narratives in Decision Aids provided a structured framework, it has not yet been formally validated. As this study focused on the feasibility and theoretical application of the taxonomy, we cannot draw conclusions about how different narrative types affect patient understanding or decision-making. Finally, we found patient narratives sometimes overlap across multiple taxonomy categories. While we used a narrative lens to help classify these ‘hybrid’ accounts, our primary focus remained on taxonomy fit rather than full-scale (thematic) narrative analysis. This reflects the practical constraints of PtDA development, where deep narrative analysis is not always feasible. Future research could focus less on categorization and more on how these mixed narratives function as a whole.

### 4.2 Innovation

Our qualitative focus on daily-life implications adds depth to what is currently communicated with BCPs about breast cancer RT side effects in both the Netherlands and Internationally –which typically emphasize describing which side effects exist, and how they can be mitigated, with little to no focus on daily impacts [27], [28], [29], [30], [31], [32]. To our knowledge, this is the first qualitative study to examine how patients live with RT side effects, providing rich insights that may inform improved risk communication.

Our findings also resonate with broader evidence of communication gaps in oncology consultations. In the Netherlands, a study of SDM in cancer care (including 886 BCPs, 27.2% of the sample) found that less than half of patients felt their values related to daily life and future plans were adequately addressed [33]. Participants reported being more frequently informed about short-term consequences of treatment (81%) than about long-term consequences (53%) [32]. Similarly, other studies show that BCPs consistently express a need for more information on how treatment side effects influence daily QoL [11], [13]. International data confirm these patterns: only 49% of patients reported their medical oncologist “always” or “very often” asked about QoL, with even fewer reporting this for nurses [15]. Together, this suggests that the impact of side effects on QoL remains under-communicated in cancer care.

### 4.3 Conclusion

This study provides insights into how former BCPs experience the impact of RT side effects on daily life, and how these experiences can be meaningfully categorized using the Taxonomy of Patient Narratives in Decision Aids. The findings highlight several QoL impacts and indicators for which additional attention should be given in the context of breast cancer RT SDM. Future research should examine how different types of narratives affect patient engagement and decision outcomes in real-world settings.

## Declaration of generative AI and AI-assisted technologies in the manuscript preparation process

During the preparation of this work the author(s) used ChatGPT (Open AI, GPT-5) in order to improve basic readability and language of the work. After using this tool/service, the author(s) reviewed and edited the content as needed and take(s) full responsibility for the content of the published article.

## CRediT authorship contribution statement

**Madeline S. Therrien:** Writing – review & editing, Writing – original draft, Project administration, Methodology, Investigation, Formal analysis, Conceptualization. **Simone M.B. Mingels:** Writing – review & editing, Formal analysis. **Rianne R.R. Fijten:** Writing – review & editing, Supervision, Funding acquisition, Conceptualization. **Daniela B. Raphael:** Writing – review & editing. **Marcel Stam:** Writing – review & editing, Funding acquisition. **Desiree van den Bongard:** Writing – review & editing, Funding acquisition. **Olga C. Damman:** Writing – review & editing, Supervision, Funding acquisition, Conceptualization. **Liesbeth J. Boersma:** Writing – review & editing, Supervision, Funding acquisition, Conceptualization.

## Funding

This study was part of a project funded by the Kwaliteitsgelden Medisch Specialisten (SKMS) [Deelprogramma Kwaliteitsproject (Sub-programme Quality Projects)].

## Declaration of competing interest

The authors declare that they have no known competing financial interests or personal relationships that could have appeared to influence the work reported in this paper.

## Appendix A Supplementary data

Supplementary material 1 Image 1

Supplementary material 2 Image 2

Supplementary material 3 Image 3

Supplementary material 4 Image 4
