# Supplementary material for: In their own words: A qualitative study of patient narratives on daily life after breast cancer radiotherapy
Source: PEC Innov. 2026 Mar 6;8:100468. doi: 10.1016/j.pecinn.2026.100468 (PMC12993325; doi:10.1016/j.pecinn.2026.100468)
Supplement: Supplementary file 1 — Supplementary material 1 [file mmc1.pdf]

Interview Guide

Q: As previously mentioned, you were asked to participate in this study because you indicated that you experienced side effects as a result of your radiation treatment. Could you tell me a bit more about the specific complaints you are experiencing?

- Do these side effects bother you a lot, a little, or moderately?
- Did you ever experience such complaints before the radiation?
- At what moments in your daily life do you encounter these complaints? How do they manifest?

Q: Looking back at your life before the radiation: were there specific things you could do then that you can no longer do because of your complaints? (For example, hobbies or other daily activities.)

- Do you experience a lot of difficulty with this?

Q: Do you have hobbies?

- Did you have the same hobbies before the radiation?
- Has the radiation affected your hobbies? Or can you still do them (in the same way)?
- How does that feel?

Q: What does your daily life look like?

- Do you work?
- How do you get to work? Do you take the car or bike?
- Do you have children?
- Do you have pets?

Q: Did you also experience complaints in the first weeks/months immediately after the radiation?

- How would you describe those complaints compared to what you experience now? Did they bother you just as much?

Q: Are there any other things we haven't discussed that you would like to share?
