# Supplementary material for: In their own words: A qualitative study of patient narratives on daily life after breast cancer radiotherapy
Source: PEC Innov. 2026 Mar 6;8:100468. doi: 10.1016/j.pecinn.2026.100468 (PMC12993325; doi:10.1016/j.pecinn.2026.100468)
Supplement: Supplementary file 3 — Supplementary material 3 [file mmc3.pdf]

Codebook: BRAVE

Data analysis method: Deductive Thematic Analysis

Code	Description
Functionality	Primarily includes:<br>- Limitations in shoulder/arm stiffness (e.g., connective tissue problems, fibrosis, muscle problems, hard chest)<br><br>And, to a lesser extent:<br>- Problems with swallowing<br>- Heart damage<br>- Lung damage<br>- Pneumonitis<br>- Bone damage and fracture
Pain	Experiences of pain caused by:<br>- Stiffness<br>- Aching<br>- Burning<br>- Stinging<br>- Scar-pain
Fatigue	Includes the experience of fatigue after breast cancer radiation therapy
Concentration	Includes the experience of concentration problems post breast cancer radiation therapy
Work	Includes experiences relating to the patients' work post breast cancer radiation therapy
Independence	Includes experiences of lack of independence post breast cancer radiation therapy.
Skin	Includes:<br>- Itching<br>- Irritation<br>- Redness<br>- Discoloration<br>- Burning<br>- Broken skin<br>- Edema<br>- Molting<br>- Red skin<br>- Blood vessel problems
Shape	Includes:<br>- Changes in the shape of the breast<br>- Unsatisfactory cosmetics<br>- Edema
