# Supplementary material for: In their own words: A qualitative study of patient narratives on daily life after breast cancer radiotherapy
Source: PEC Innov. 2026 Mar 6;8:100468. doi: 10.1016/j.pecinn.2026.100468 (PMC12993325; doi:10.1016/j.pecinn.2026.100468)
Supplement: Supplementary file 4 — Supplementary material 4 [file mmc4.pdf]

Data Analysis: Code Tree & Taxonomy Classification				
**Theme 1: Pain**				
**Subtheme**	**Interview #**	**Narrative**	**Content**	**Evaluative valence**
**1.1: Pain with touch**	Interview 2	*"...and that was so annoying, because when I laid on my [irradiated] side [I felt pain]."* **(P2, Q1)**	Experiential	Negative
	Interview 3	*"... it was also after a few weeks that I actually started experiencing pain there. Especially when someone touched it, you know. My therapist, as a sort of joke—not as a joke but just to see—pressed on my ribs, you know. ... So he touched it with a bit of pressure and then I really recoiled. I usually have a high pain threshold."* **(P3, Q1)**	Experiential	Negative
	Interview 6	*"Yes, it basically turns into light scar tissue and becomes hard, and if someone hugs you a bit too firmly, then you feel that — and that doesn't go away, I still have that.But that probably won't ever go away."* **(P6, Q1)**	Outcome	Neutral
		"No, yeah, you also have to be careful not to bump it into anything, because if that does happen, then I do have some lingering pain from it, but otherwise, not really." **(P6, Q2)**	Outcome	Neutral
	Interview 9	*"Uh, it's not that I can't do certain things, but if someone... if someone hugs me and it's too tight, then... ouch."* **(P9, Q1)**	Outcome	Neutral
		"It's just that, yeah, a regular bra — I can't... that doesn't work. I always wear those elastic camisoles. It's not comfortable. It really doesn't feel good." **(P9, Q2)**	Experiential	Negative
		"No, only when sleeping. If I lie on my [irradiated] side, then I put my hands underneath, then... that's quite uncomfortable." **(P9, Q3)**	Experiential	Negative

	Interview 10	*“If I go for a check-up now and I have to do the mammogram... oh... oh. ... Then I think, ‘Woah, you really need to find a different way for that, because it really hurts.’ I still have to go for one of those mammograms twice more, but every time I think... I’ll postpone it. Of course, that’s silly, but it really does hurt, yes.”* ***(P10, Q1)***	Experiential	Negative
		“Yes, yes, yes. When I lie on my stomach in bed [its sensitive]” ***(P10, Q2)***	Experiential	Negative
	Interview 12	*“... so you have pain from being touched, because that persists”* ***(P12, Q1)***	Outcome	Negative
	Interview 14	*"Yes, sleeping was very difficult for a while, especially lying down. Because I had a lot of discomfort with sitting and lying down.”* ***(P14, Q1)***	Outcome	Negative
**1.2: Chronic discomfort**	Interview 2	*“No, no. Then it settles again. But that feeling in the ribs remains. Not pain, but the feeling that... it just doesn’t feel right.”* ***(P2, Q2)***	Experiential	Negative
		“But if you really look at it as: what causes discomfort? Then it’s from here to the ribs. So from just past the armpit to the ribs. That remains unpleasant” ***(P2, Q3)***	Outcome	Negative
	Interview 8	*“I thought, ‘Oh, it will probably fade afterwards,’ but it really remains a sensitive spot. The area that was irradiated, actually.”* ***(P8, Q1)***	Outcome	Negative

	Interview 10	*"I've finished it, I've done the radiation, but it just remains painful. It's still painful now. The whole breast is still painful. It's not like you think, 'I have a day [without pain]...' 'Pain' is a big word, but I haven't had a day without discomfort from it — that's how I should put it."* ***(P10, Q3)***	Experiential	Negative
	Interview 11	*"You never normally feel this. Normally, you don't feel your limbs at all. Not even when you move them. But now you do feel them. They're basically present all day—sometimes more, sometimes less, but they're always there. And you feel that."* ***(P11, Q1)***	Experiential	Neutral
	Interview 14	*"Yes, it feels better than after the surgery. But it's not like it was before, you know. I'm always aware of it. So I'm constantly reminded of it. It's still a bit swollen in that area."* ***(P14, Q2)***	Experiential	Neutral
		"I still have discomfort in my ribs." ***(P14, Q3)***	Outcome	Negative
**1.3: Sports**	Interview 3	*"... I especially noticed [the pain] when exercising. And that only came later, in the long term — I think even after a year."* ***(P3, Q2)***	Outcome	Neutral
	Interview 6	*"Yes, for example, diving into the water. Yes, because that really hurts, and then I'm in pain for quite a while afterward."* ***(P6, Q3)***	Experiential	Negative
**Theme 2: Arm functionality**				
**Subtheme**	**Interview #**	**Narrative**	**Content**	**Evaluative valence**

**2.1: Limitations in movements**	Interview 2	*"But you know, I've always had an office job. And now I'm retired. So I can completely adjust my movements to suit myself. But if I still had a different kind of job now — one where you really need to reach a lot and use your arms — then I think I would definitely be struggling with it." (*Moderator: And that's different because you're retired now?) *"Yes, now I don't have to. It's like taking the elevator instead of the stairs — that's how I see it. I can still take the stairs, sure. But why would I? That's how I look at it."* ***(P2, Q5)***	Outcome	Neutral
		"And what I can't do anymore— I do aquarobics every now and then, and then we have to use a ball. And when I do something, I like to really do my best. But I can't anymore, because when I reach for a ball, I can have pain for the whole afternoon." ***(P2, Q6)***	Outcome	Negative
		"... only in the water, playing a ball game... and that's because I don't take it into account. Out of enthusiasm, you actually forget about it. Then I end up having pain." ***(P2, Q7)***	Outcome	Negative
	Interview 3	*"Especially that my major chest muscle was really tight. So I did experience some movement restrictions there and also some pain when trying to lift it. But it's not completely gone — I can move my arm up again on the other side. But I still felt it myself, especially in the area where the breast radiation was done."* ***(P3, Q3)***	Outcome	Negative

	Interview 8	*“No, it’s not that I can’t do them, but in some areas I am more often—well, “limited” is such a big word—but you do notice it. For example, if I have to take something out of the cupboard, everything feels really sensitive.”* ***(P8, Q3)***	Outcome	Negative
	Interview 9	*“Well, when you reach backward for something, you can really feel that muscle pulling, like that muscle tightens up.”* ***(P9, Q4)***	Outcome	Negative
	Interview 10	*“Yes, a muscle in my arm feels irritated. I had to look for a different job, you know, something like that. But then I think… …yeah, well, it’s all not that… …not that bad. I can handle all of that. I worked in the [redacted] industry for a long time. And after that, in the [redacted] business. But that was too physically demanding. I couldn’t handle that kind of work anymore.” (P10, Q4)*	Outcome	Negative
		“Yeah, I can’t straighten my arm anymore, I can’t lift heavy things anymore. Yeah, I can do it, but then… [it’s painful].” ***(P10, Q5)***	Outcome	Negative
		“Yeah, in the beginning, everything is really hard. I had to completely rearrange the kitchen. You just have to find your way a bit. That’s just how it is, I think.” ***(P10, Q6)***	Outcome	Negative
	Interview 14	*“Yes, when I’m exercising, I definitely notice it. With certain exercises, that shoulder is limited. They’re really strange movements, because if I just calmly raise my arm, that’s fine. But if I have to make an unexpected movement, especially a twisting one—like a front crawl in the pool—that just doesn’t work. But just swimming is okay.” (P14, Q4)*	Outcome	Negative

**2.2 Loss of strength**	Interview 3	*"Yes, I definitely had less strength to get out of my arm. And I really liked cycling—both mountain biking and road cycling. Mountain biking still goes pretty well. I really like going to Limburg to mountain bike. There, you sit more upright on your bike. But with road cycling, you lean forward a bit more, and that's where I've really, to my great frustration, never been able to find a comfortable position to ride long distances again."* ***(P3, Q4)***	Outcome	Negative
	Interview 11	*"Yes. I bought a smaller car, because of the door. Very simple things. We have quite a big car. But now I drive like I used to in the past, a Fiat 500, because I just can't handle the door and things like that. You really notice that. Yes, you don't have that strength anymore. That's very difficult."* ***(P11, Q2)***	Outcome	Negative
		"Look, when I used to put the bike outside and had to hold a door while doing that, that was no problem at all. Now I have to think about it, because how do I manage that? Because I simply don't have enough strength in my arms anymore." ***(P11, Q3)***	Outcome	Negative
**Theme 3: Changes in breast appearance**				
**Subtheme**	**Interview #**	**Narrative**	**Content**	**Evaluative valence**

**3.1 Managing a new appearance**	Interview 2	*"No, no, no, no. That's not something I have a problem with. Okay—well, I did have a lot of issues with it in the beginning, you know? Because then you have one breast over here and another one over there. I also have a granddaughter who's six. And she said it very bluntly once—we went swimming together, because I really love swimming. Just yesterday, I swam for three hours. And she said, "Grandma, you have one small breast and one big breast!" I didn't mind, really. I mean, it's just a child... But the whole hassle with padding a bra and all that—that I truly hated. By the end, you don't even know what you're wearing anymore. I used to have fairly large breasts—yes, I'm a big woman. And then you have to match everything to the bigger one, and you think, "Yeah, but this just doesn't look right at all anymore."* ***(P2, Q8)***	Experiential	Negative
		"I can totally understand that some women choose to have a lot removed instead. So it doesn't look uneven. Because the hassle you have to go through just to feel a bit balanced — it should look somewhat equal. So, I found that really frustrating, but luckily it was resolved. Then the plastic surgeon said, "Oh, that looks nice," but she also warned me, "Keep in mind the left, the radiated breast, that one won't do anything anymore." That means, it stays as it is. So maybe you'll come back for the right breast later, because it will still sag. But the left one won't. It's static, it's firm, it doesn't move anymore. So from that, you can see that the radiation definitely has an effect on your breast." ***(P2, Q9)***	Outcome	Negative

		"So you see, for example… I used to have round breasts. Like apples. And this one was corrected and has stayed like an apple. But this one—there's nothing left here. It was also located here, so they had to do all kinds of tricks and techniques to make it look somewhat decent. And with the scar—I still have a hard patch here, a firm edge. And they can't do anything about it, because according to the oncology team and the plastic surgeon, it's a result of the radiation, which caused it to harden." ***(P2, Q10)***	Outcome	Negative
	Interview 4	*"But because of that weird bra construction, especially with the harness, you end up with completely flattened breasts. And that changes the way you dress. I stopped feeling attractive. But now I've taken a tip from someone: what that harness does, just wear it at night. Don't wear those things during the day. And if I'm wearing a sweater or something and it doesn't show, then sometimes I'll use one of those… you know, those pads with little bumps on them. And the bumps help keep the fluid moving. If no one sees it, then it doesn't bother me. There are all these little things — but added up… Yeah, all together, it's just a hassle." (P4, Q1)*	Experiential	Negative
	Interview 10	*"Yeah, this breast is totally mis shaped, that I'm almost a cup and a half smaller, because of the radiation, whereas before, it was just even, you know?"* ***(P10, Q7)***	Outcome	Negative

	Interview 14	*"Maybe if it just looked really neat, I wouldn't mind so much. But the way it is now, yeah, I do find it a bit of a thing." **(P14, Q5)***	Experiential	Negative
**3.2: Partner support**	Interview 4	*"Look, you have to imagine: when I was told I had cancer, my husband came home and, very lovingly, said: "Even if they have to remove your breast, I still love you. So don't worry about that—we'll get through this together." That meant everything. He had no idea how important that was for me. It really was sweet. Especially because, well, he was always kind of a "breast man."" **(P4, Q2)***	Experiential	Positive
	Interview 8	*"Everything on that side just looks different. So yeah, it's different for everyone, but I've simply accepted it — and again, my sweet husband as well. That really makes a big difference overall. So for me, yes, the difference is there, but... well, the cancer is gone." **(P8, Q4)***	Experiential	Neutral
	Interview 10	*"And I've kind of made peace with it. My husband — he's like, you know, he doesn't need any of it. He just says, "You're perfect the way you are." **(P10, Q9)***	Experiential	Positive
**Theme 4: Skin irritation**				
**Subtheme**	**Interview #**	**Narrative**	**Content**	**Evaluative valence**

**4.1: Needing aftercare**	Interview 3	*"Yes, I did have some skin complaints. The tissue swelled up. I had some skin issues, so some discoloration appeared. The skin started to peel a little, but that was resolved with an ointment. It went away after a few weeks."* ***(P3, Q5)***	Outcome	Neutral
	Interview 4	*"That was really tough, and I had to use bandages. In the end, it stayed open for about ten weeks."* ***(P4, Q3)***	Outcome	Negative
		"… because the skin was open, so I had to wear a sort of bandage. I had really good dressings, which I got from the radiation department in [redacted]. They also gave me a very tight-fitting T-shirt they had designed themselves." ***(P4, Q4)***	Outcome	Neutral
		"… and this past January—more than a year later—it became sensitive again, turned red again. … And then I have to apply cream myself, but that's not good for the bra, and I have to wear those corsets…I really hate them." ***(P4, Q5)***	Outcome	Negative
	Interview 5	*"When I look at the so-called damage I had quickly developed—yes, the skin did become a bit red. That had to be treated with cream, which I just did routinely, so to speak"* ***(P5, Q1)***	Outcome	Neutral

	Interview 6	*"Yes, at one point I did notice something — when I washed over it with soap, I could feel a slight stinging. So I immediately started applying that Alhydran cream, yes, just like the radiotherapists recommend during the intake. And after that, I didn't have any trouble at all. Nothing."* ***(P6, Q4)***	Outcome	Neutral
	Interview 10	*"And what I found really upsetting during the radiation was that I had to start using bandages, because my skin was so damaged from those thick adhesive plasters. They give you one to take home from here, and then you have to call because...then they give you a number to call, but actually, there's nothing they can really do either. I found that very frustrating. And then that woman starts telling me how expensive those plasters are."* ***(P10, Q10)***	Outcome	Negative
	Interview 12	*"Yes, because they had told me in advance that my skin might turn red. And it did sometimes, but then I would just apply some cream really well. And I did that faithfully."* ***(P12, Q2)***	Outcome	Neutral

**4.2: Unsatisfactory cosmetics**	Interview 4	*“Yeah, so they didn’t talk about the skin peeling at all. And the first time I wanted to show it, I felt a bit like some kind of fairground attraction. They’d lift up your breast, and if they thought, “oh yes, of course,” then—well, actually, it made sense—they already had the dressings ready. And yeah, I just hadn’t really thought that something like that could happen.”* ***(P4, Q6)***	Experiential	Negative
	Interview 10	*“Yes, I was really burned. I was actually black under the armpit. It really hit hard, I have to say. Especially that last week.”* ***(P10, Q11)***	Experiential	Negative
		“Yes, and also the skin itself, including the nipple. I had a breast reduction, and the nipple was removed and repositioned. But also the skin—it feels a bit like leather. I don’t know how else to put it, but it’s not very sensitive; it’s kind of like leathery skin. Yes, and also the color. It’s white. Just a white nipple.” ***(P10, Q12)***	Outcome	Neutral
	Interview 14	*“And the scar really changed afterwards — it became very hard and bumpy. It actually looked much better in the beginning. Yeah, the outcome… the cosmetic outcome, I’d say, is not really good.”* ***(P14, Q6)***	Outcome	Negative

**4.3: Sun avoidance**	Interview 3	*"I remember that we had planned a vacation a month after the radiation treatment. That was just in time for it to have healed. But I was extra careful with the sun. I didn't go out in the sun or anything."* ***(P3, Q6)***	Outcome	Neutral
	Interview 5	*"And after my last radiation treatment, let's say about three weeks later, we went on vacation. We went to Greece. I had asked beforehand whether that would be a problem. I just had to be careful, wear a shirt over it and stay in the shade. I had to be mindful of that anyway."* ***(P5, Q2)***	Outcome	Neutral
	Interview 12	*"You're not supposed to be in the sun, because yesterday I could really feel it — now it's gone... I put lotion on twice, but it felt like I had all these tiny little bumps, a bit like sandpaper... but now it's gone again. But I think it was because the sun was out yesterday. You can try to stay out of the sun, but there are always moments where you can't avoid it."* ***(P12, Q3)***	Outcome	Neutral

**4.4: Difficulties with intimacy**	Interview 4	*"But then, you know, you have to get used to a scar like that, and the sentinel node scar, and all those marks—and actually, I got used to it pretty easily. But when you've got such a vulnerable wound, an open breast like that, you can't be physically close. So that's... it's just... well, I was still a cheerful person during that time—still am—but it wasn't pleasant. I remember thinking, "I'd give anything... I'd give anything if it would just heal." It was such a situation. Eventually, after ten weeks, it closed up. But that's a long time."* ***(P4, Q7)***	Experiential	Negative
	Interview 12	*"Well, the touch, like I said... So when my husband touches my breast, this one is less sensitive. Also the... yeah, I'm a bit embarrassed to say it... the nipple is also less sensitive."* ***(P12, Q4)***	Experiential	Negative
**Theme 5: Fatigue**				
**Subtheme**	**Interview #**	**Narrative**	**Content**	**Evaluative valence**
**5.1: Fluctuating energy**	Interview 2	*"And I was really tired. But it was a very strange kind of fatigue — at least, for me personally. It wasn't the kind of tiredness like, "Oh, I've done a lot, and now I'm tired." No, it was more like, "Oh..." — it would just come over you. And then, "Wow, I need to lie down now.""* ***(P2, Q11)***	Experiential	Negative

	Interview 6	*"Yeah, you just don't see it coming. It's not the kind of fatigue you can just push through. Like, "Oh, I didn't sleep well last night — I'll just go to bed early tonight and feel better tomorrow." No, I'd be struggling with it for a whole week. And it would take me a long time to recover from it. So yeah, it's hard to explain, but it's the kind of tiredness you can't just shake off. It's not something that's gone the next day. And I also noticed that this year, this winter, I felt it even more. Normally, the darker days don't really affect me. But this time, I felt even more exhausted. I'd get through the day, go to bed, sleep until seven in the morning — and still feel wiped out. That's never happened to me before, but it really hit me this past winter."* ***(P6, Q5)***	Experiential	Negative
		"Because I also notice that when I'm really tired, the neuropathy tends to come back a bit. I can still feel it in my feet sometimes. And then I think, "Ah yes, I really am exhausted." Often that's when I end up taking a nap in the afternoon — just suddenly and then I can go on again for a few days or even weeks." ***(P6, Q6)***	Outcome	Neutral

		"No, I don't really avoid anything. I still have young children and, well, just a family. And that's what keeps you going. But if we have, say, a party or something in the evening, then I'll make sure to take a proper nap in the afternoon, and that works out fine. So no, it's not a problem at all." ***(P6, Q7)***	Experiential	Neutral
	Interview 8	*"And on top of that comes the fatigue. The fatigue really fluctuates — it comes and goes — but it's definitely there. I notice that... actually throughout the whole process, and even afterwards, the fatigue has remained."* ***(P8, Q5)***	Outcome	Negative

		“Well, it really depends. What are you doing, you know? How does your day start? For example, I work as a [redacted]… But I notice that when I start early in the morning, like at 7:30 or 8:00, and I do my shift, then by around 2:30 or 3:00 I’m just glad it’s over. I think, “Okay, that’s done.” And if I can lie down for even just half an hour after that — with no stimuli — I can handle things a bit better. Sometimes that works out, but sometimes it just doesn’t at all. Then I think, “I still need to do this, and I still need to do that…” And if I have an afternoon shift, like today — well, right now I feel okay, but later it could suddenly hit me. I can just crash out of nowhere, and then I think, “Oh no, I still have to keep going until 9:00 tonight…” And yeah, that can be tough sometimes. I just feel so tired.” ***(P8, Q6)***	Experiential	Negative
	Interview 12	*“That… immense fatigue, it's not regular tiredness. Like after a day of work — no, it’s a kind of exhaustion that just knocks you out. And it hits you at moments when you're not really expecting it. One day you're fine, and the next… Even now, I still experience a certain fatigue — like behind my eyes. But then I wonder, is it hay fever? You never really know what exactly causes it. I still sleep almost every day.”* ***(P12, Q5)***	Experiential	Negative

		"But even then, that fatigue does come. [My husband] often says, "Shouldn't you lie down for a bit before…" or in between things… He can see it in me. I wouldn't notice it myself, but he says, "Why don't you lie down for a bit?" And then I do. Often just half an hour or even fifteen minutes is enough, and then I feel better again." ***(P12, Q6)***	Experiential	Neutral
	Interview 13	*"Yeah… then [the impact is] really about the housework, right? If I feel like… I'm tired now. Well, then I just sit down for a bit. I take a rest. And after a while, I can get back to it again."* ***(P13, Q1)***	Experiential	Neutral
**5.2: Stamina**	Interview 8	*"My breathing — that's definitely an issue. Before all this, I used to smoke. But ever since I started treatment, I quit completely — just stopped — and that's been fine. But still, I often feel as if I've just smoked an entire pack of cigarettes."* ***(P8, Q10)***	Experiential	Negative
		"Yes, exactly. That's just what we discussed — because of the fatigue and my condition, well, that's really what comes through the most. Because of that, every day you have to reassess how you feel, what you can handle, and what you're going to do. And also, you have to accept it, actually. It's not always easy, but I think it's just part of everything now." ***(P8, Q11)***	Experiential	Neutral

	Interview 9	*"Yeah, because before I didn't experience this [fatigue] at all. Not at all. Sure, of course you're tired when you climb the stairs, but now, for example, if I have to rush to the station, I can't catch my breath anymore."* ***(P9, Q6)***	Outcome	Negative
		"Well, I'm currently in rehabilitation, and I do notice—yes, that's what rehab is for, to try and build up my condition. And even the simplest exercises, if you do them a few times, you just can't catch your breath." ***(P9, Q7)***	Outcome	Negative
		"Yeah, it really happens, like when we walk to the store and we walk a bit too fast, then I say, "Whoa, hold on a second."" ***(P9, Q8)***	Experiential	Negative
	Interview 11	*"And in the beginning, I felt very weak, and the oncologists did say that you also experience that with radiation treatment. For example, [before treatment] I could easily walk 10 kilometers, but if I had to climb a staircase, I would be completely exhausted."* ***(P11, Q6)***	Outcome	Negative
		"In the beginning, I did have that with the radiation treatment. I can cycle a lot, and I really enjoy cycling. But back then, I couldn't cycle a single meter. I was exhausted. Now I can cycle 60 kilometers, it doesn't matter. I really had to rebuild my strength, like cycling to Albert Heijn and back." ***(P11, Q7)***	Outcome	Negative
**Theme 6: Concentration**				

Subtheme	Interview #	Narrative	Content	Evaluative valence
**6.1: Difficulty reading**	Interview 2	*"You know... Before, I could read a whole book in one sitting. And now I notice that even one chapter is a struggle. And that's something I'm really relearning. I'm still working on that reading. Reading is still the most beautiful thing to me. Yes, it just is. You can create your own world. And I do find that a shame."* ***(P2, Q16)***	Outcome	Negative
	Interview 13	*"But when I read, I really do have to... I actually have to sit down for it, and I really have to be able to concentrate. Because otherwise I'll be reading and at some point I'll think... "Wait, what was that again?" And then I have to go back a page."* ***(P13, Q6)***	Outcome	Neutral
**Theme 7: Comparing to pre-treatment self**				
**Side Effect**	**Interview #**	**Narrative**	**Content**	**Evaluative valence**
Changes in breast appearance	Interview 2	*"So you see, for example... I used to have round breasts. Like apples. And this one was corrected and has stayed like an apple. But this one—there's nothing left here. It was also located here, so they had to do all kinds of tricks and techniques to make it look somewhat decent. And with the scar—I still have a hard patch here, a firm edge. And they can't do anything about it, because according to the oncology team and the plastic surgeon, it's a result of the radiation, which caused it to harden."* ***(P2, Q10)***	Outcome	Negative

	Interview 8	*"Everything on that side just looks different. So yeah, it's different for everyone, but I've simply accepted it — and again, my sweet husband as well. That really makes a big difference overall. So for me, yes, the difference is there, but... well, the cancer is gone."* ***(P8, Q4)***	Outcome	Neutral
	Interview 10	*"Yes, this breast is so deformed that it's now almost a cup and a half smaller, because of the radiation, while in the beginning, they were the same, you know?"* ***(P10, Q8)***	Outcome	Negative
Fatigue	Interview 8	*"Well, you know... sometimes it's frustrating. You just want to get back to normal again. Look, of course I'm incredibly grateful for everything and everyone and how it all went. Definitely. But in between, it's still frustrating. You think, damn it. I just want to have the normal back a bit."* ***(P8, Q9)***	Outcome	Negative
	Interview 9	*"Yeah, because before I didn't experience this [fatigue] at all. Not at all. Sure, of course you're tired when you climb the stairs, but now, for example, if I have to rush to the station, I can't catch my breath anymore."* ***(P9, Q6)***	Outcome	Negative

		"…I mean, of course I worked before and I would rather be fitter and just work. So you have to come to terms with that. And that is already difficult. It's not something you've always done, and now you can't anymore." ***(P9, Q5)***	Outcome	Negative
	Interview 11	*"Yes, I've definitely become much more tired. I don't really know if it's because of the radiation treatment. That's very hard to say. But where I used to be able to work for days on end and needed very little sleep, that's different now. Yes, if I've had a really full day, then in the evening… And when I get home, I'm really completely drained. Before, I could still do the gardening and the laundry, but now I have to pace myself. And sometimes I really have to say, okay, that's enough, it's over. And then, for example, if I relax, lie down on the bed, watch TV, I recover pretty quickly."* ***(P11, Q5)***	Outcome	Neutral
		"And in the beginning, I felt very weak, and the oncologists did say that you also experience that with radiation treatment. For example, [before treatment] I could easily walk 10 kilometers, but if I had to climb a staircase, I would be completely exhausted." ***(P11, Q6)***	Outcome	Negative
	Interview 13	*"Yeah, I just can't handle it. Before, I was always busy — doing like ten things at once. Yeah, that just doesn't work anymore."* ***(P13, Q2)***	Outcome	Negative

Concentration	Interview 2	*"And in the beginning, [my concentration] was quite bad. I really had this feeling of, "What happened?" And I'm someone – I used to [redacted] – and I'm very detail oriented. So really the little things, I had to keep track of them. And that was one of the hardest things when I went back to work after three months: that I had lost that feeling. And it's gotten much better, but it will never be the same again."* **(P2, Q15)**	Outcome	Negative
		"You know... Before, I could read a whole book in one sitting. And now I notice that even one chapter is a struggle. And that's something I'm really relearning. I'm still working on that reading. Reading is still the most beautiful thing to me. Yes, it just is. You can create your own world. And I do find that a shame." **(P2, Q16)**	Outcome	Negative
	Interview 10	*"But there are things... that I used to...Things I used to remember more easily, somehow. That I now... I now need a pen and paper for that sometimes."* **(P10, Q13)**	Outcome	Neutral
	Interview 13	*"I used to just sit out in the open office... yeah, yeah. Hopefully that will come back someday. But right now, just when I'm in that booth... Still with my colleagues on the same floor, of course... Yeah, then I can concentrate better."* **(P13, Q5)**	Outcome	Negative
**Theme 8: Acceptance**				

Side Effect	Interview #	Narrative	Content	Evaluative valence
Pain	Interview 2	*“Let me put it this way: it’s annoying. But I classify it as one of the minor ailments You have these things—you have to deal with them. Or... they have consequences, and then you have to do something about them. I talked about this with [redacted] as well. Yes, and [my radiation oncologist] also said, “I wouldn’t know what we could do about it right now.” And then I say: yeah, it's just a minor ailment.”* ***(P2, Q4)***	Experiential	Neutral
	Interview 12	*“You also learn to... you start accepting things. Eventually, you even start to accept pain.”* ***(P12, Q2)***	Experiential	Neutral
Changes in breast appearance	Interview 1	*“No, it’s a bit of a shame, but I don’t suffer from it. Maybe that’s because... Well, you know, when you’re 70, you don’t have to look perfect. But it’s different when you’re 40 or 30 or whatever, you still want to look good, of course.”* ***(P1, Q1)***	Experiential	Neutral
	Interview 8	*“Everything on that side just looks different. So yeah, it’s different for everyone, but I’ve simply accepted it — and again, my sweet husband as well. That really makes a big difference overall. So for me, yes, the difference is there, but... well, the cancer is gone.”* ***(P8, Q4)***	Experiential	Positive

	Interview 11	*"I had surgery… differences in the size of the breast, everything that comes with it. And the scars, that's all fine. For me, it's really not a big deal. If you're 45, 40, or 35… …then I think it has a much bigger impact."* ***(P11, Q4)***	Experiential	Neutral
**Theme 9: Work**				
**Side Effect**	**Interview #**	**Narrative**	**Content**	**Evaluative valence**
Fatigue	Interview 1	*"I started working more, and I was tired, and I had stomach pain during the radiation. I think it was right around that time. But that wasn't really talked about, so I ended up working even less. At that moment, a colleague noticed I was struggling, and I kept telling myself: don't complain. Then I talked to my manager, and he said, you know what, just come in, and if you can't do much, then don't do much. But that didn't really work out. So what I want to say is, that was probably the hardest time — going to work when I was tired, having stomach pain, headaches, and maybe also emotional stuff because I was so tired."* ***(P1, Q2)***	Experiential	Negative

		*"Actually, I shouldn't have done that either. Because I went home with headaches, stomach pain, and I was basically dragging myself home. And it wasn't in me to say, just call in sick. And I think, maybe they're not allowed to give that advice, but I almost think you just have to take the time for yourself. That's what I think, when you're undergoing radiation treatment. But many people take it very lightly, and then you think, don't complain, it's not that bad. And you only really notice the impact when you do go to work." **(P1, Q3)***	Experiential	Negative
		*"No, I think I was able to organize my life more [after retirement]. If I needed to lie down, I could. That pressure was gone." **(P1, Q4)***	Experiential	Positive
	Interview 2	*"I worked fewer hours for quite a long time because I just couldn't manage. When I got home, well... that was it. I really couldn't do anything else. So it was really... and at a certain point, you noticed that you just couldn't anymore. You had to kind of push yourself not to collapse, so to speak. Yes. I also used to work a lot alone, especially during busy times like the annual accounts period. Working late into the night, for example. But now I didn't dare to do that. Because if I wasn't... distracted, I would just fall asleep." **(P2, Q12)***	Experiential	Negative

	Interview 4	*"But I am retiring because I find [the side effects] unpleasant. I feel a bit not good enough. And if I think, 'What are we talking about? What's the topic?' Yes, I've missed quite a lot in the past year. Well, I find that awful. Then I lose my foundation of certainty. And yes, that's still not really recovered. So stress and fatigue and concentration…"* ***(P4, Q9)***	Outcome	Negative
	Interview 8	*"And I just find it really hard to say at work, 'Just don't give me those afternoon shifts. Let me do the morning shifts. Then I won't bother you in the evenings.' Not that I'm bothering them, but I'm often so tired that I think, 'Oh, how am I going to manage everything?' So I often put a bit of pressure on myself. Yes, I do find that difficult"* ***(P8, Q7)***	Experiential	Negative
		"I have lovely colleagues, and then I think [if I have to work different shifts to account for my fatigue], 'Ah, they have to cover that shift.' So I end up feeling a bit guilty. So yes, that's how it is." ***(P8, Q8)***	Experiential	Negative
	Interview 9	*"…I mean, of course I worked before and I would rather be fitter and just work. So you have to come to terms with that. And that is already difficult. It's not something you've always done, and now you can't anymore."* ***(P9, Q5)***	Outcome	Negative

	Interview 14	*"I just have the feeling that now everyone's expectations are back to how they were before. Also at work, for example. There are new colleagues who don't even know I've been ill. And sometimes it just doesn't work. I find it really difficult to say then…'Now it just doesn't work.' Or 'Now I need to call in sick.'"* ***(P14, Q7)***	Experiential	Negative
Concentration	Interview 2	*"Working was really hard. In the evenings, I was completely exhausted. I was really dead tired because I had to put so much effort into maintaining my concentration."* ***(P2, Q14)***	Experiential	Negative
	Interview 4	*"But I am retiring because I find [the side effects] unpleasant. I feel a bit not good enough. And if I think, 'What are we talking about? What's the topic?' Yes, I've missed quite a lot in the past year. Well, I find that awful. Then I lose my foundation of certainty. And yes, that's still not really recovered. So stress and fatigue and concentration…" (P4, Q9)*	Outcome	Negative
	Interview 10	*"Yes, I had… Yesterday I really had one of those days [where I couldn't concentrate]. I had slept badly too. And then I start asking things that I actually know. And then I think, I do know it. But at that moment, it just doesn't come to me. And then I think, yeah… that's strange, isn't it?"* ***(P10, Q12)***	Experiential	Negative
	Interview 13	*"Because I've also noticed that I have concentration problems. Sometimes with memory too — both short-term and long-term. Just concentrating in general. So that's also annoying at work."* ***(P13, Q3)***	Outcome	Negative

		"And I do call center work at [redacted]. But we also have these little booths. So you're completely isolated in there. And now I actually always sit in a booth. Because I constantly hear outside noise Or there's something going on in the open office, then I have to close the door. I just can't handle it anymore." ***(P13, Q4)***	Experiential	Negative
		"I used to just sit out in the open office... yeah, yeah. Hopefully that will come back someday. But right now, just when I'm in that booth... Still with my colleagues on the same floor, of course... Yeah, then I can concentrate better." ***(P13, Q5)***	Outcome	Negative
